# Correlation Between Social Media Posts and Academic Citations of Orthopaedic Research

**DOI:** 10.5435/JAAOSGlobal-D-20-00151

**Published:** 2020-09-01

**Authors:** Dafang Zhang, Brandon E. Earp

**Affiliations:** From the Harvard Medical School, Boston, MA (Dr. Zhang and Dr. Earp), and the Department of Orthopaedic Surgery, Brigham and Women's Hospital, Boston, MA (Dr. Zhang and Dr. Earp).

## Abstract

The objectives of this study were to quantify adoption of social media for the dissemination of original research in orthopaedic research and to determine the correlation between academic citations and social medial posts among recent orthopaedic publications.

**Methods:**

An Internet-based study was performed of 835 articles from three orthopaedic journals from 2018 to 2019. The number of academic citations for each article was determined using Google Scholar and Web of Science. The number of social media posts was determined using Twitter. The correlation between academic citations and social media posts was calculated using the Spearman rank correlation coefficient.

**Results:**

The average number of academic citations per article was 4.6 on Google Scholar and 2.6 on Web of Science. The average number of social media posts per article was 3.6, which consisted of 1.1 tweets and 2.5 retweets. The number of academic citations per article was weakly correlated with the number of social media posts, tweets, and retweets.

**Discussion:**

There is a positive relationship between social media posts and academic citations of recent orthopaedic research. Use of social media differs among journals and authors, which may represent opportunities to leverage social media platforms to more effectively dissemination novel research findings.

The use of social networking and microblogging platforms for novel information in orthopaedic surgery has risen in recent years among patients and physicians alike.^1,2,3,4,5,6^ Web-based social media platforms are increasingly used to share and disseminate original scientific research. Many academic journals have adopted Twitter profiles to have a greater social media presence, both within the academic community and the general public.^7,8^ Currently, Twitter is the most commonly used social media platform for the dissemination of medical research, accounting for more than 80% of online posts of musculoskeletal research,^9^ with more than 300 million active monthly users.^10^

Quantitative analyses of social media posts of orthopaedic surgery research publications are limited. Furthermore, the relationship between social media posts of orthopaedic surgery research and conventional metrics of research impact, such as academic citation, is not well understood.

The objectives of this study were (1) to quantify adoption of social media for the dissemination of original research by three high-impact orthopaedic surgery journals and (2) to determine the correlation between academic citations and social media posts among recent orthopaedic surgery research publications. Our null hypothesis was that no correlation between academic citations and social media posts exists among orthopaedic surgery research publications.

## Methods

An Internet-based correlation study was performed without human subjects, and thus, institutional review board approval was deferred. This study comprised all original scientific research articles from three high-impact orthopaedic surgery journals, *Clinical Orthopaedics and Related Research* (CORR), the *Journal of the American Academy of Orthopaedic Surgeons* (JAAOS), and the *Journal of Bone and Joint Surgery* (JBJS), published in printed volumes from January 2018 to December 2019. Editorials, commentaries, review articles, technique articles, short reports, case reports, conference proceedings, and errata were excluded. A final sample of 835 original full-length scientific research articles, including 210 articles from CORR, 177 articles from JAAOS, and 448 articles from JBJS, were included for analysis.

The number of academic citations for each article was determined using Google Scholar and Web of Science, both web-based platforms that index research publications and metadata from multiple databases across academic disciplines. Social media posts of each article were assessed using Twitter, a social networking platform with over 300 million active monthly users.^10^ The total number of social media posts on Twitter was determined for each article. The total posts were subdivided into original tweets and retweets. Moreover, social media posts were assessed for official tweets by the publisher, journal, or national organization or tweets by an author. All data were collected between April 1, 2020, and April 3, 2020.

Descriptive statistics for academic citations and social media posts were calculated. The correlations between academic citations and social media posts were calculated using the Spearman rank correlation coefficient and depicted graphically to show pairwise relationships. Comparisons of social media utilization among journals were performed using analysis of variance (ANOVA). Citation discordant publications, defined as publications with both academic citations and social media posts, but with at least 10 times more academic citations than social media posts or vice versa, were studied as a particular subset of interest. The standard significance criteria of α = 0.05 was used.

## Results

The number of academic citations and social media posts were determined for 835 orthopaedic surgery research publications (Table 1). The average number of academic citations per article was 4.6 on Google Scholar and 2.6 on Web of Science. The average number of social media posts per article was 3.6, consisting of 1.1 tweets and 2.5 retweets. There were on average 0.3 official tweets and 0.03 author tweets per article. Earlier publication was moderately correlated with a greater number of citations on Google Scholar (ρ = 0.61, *P* < 0.0001) and Web of Science (ρ = 0.59, *P* < 0.0001). Publication date had no significant correlation with the number of social media posts (*P* = 0.8).

**Table 1:** Number of Academic Citations and Social Media Posts of Orthopaedic Research Publications From 2018 to 2019 (Mean ± SD)

	Academic Citations (Google Scholar)	Academic Citations (Web of Science)	Twitter Posts	Tweets	Retweets	Official Tweets	AuthorTweets
CORR	3.9 ± 4.5	2.5 ± 2.8	4.5 ± 6.3	1.2 ± 1.1	3.3 ± 5.6	0.7 ± 0.8	0.02 ± 0.14
JAAOS	2.5 ± 2.9	1.2 ± 1.7	2.3 ± 9.6	0.9 ± 1.0	1.4 ± 9.4	0.03 ± 0.21	0.02 ± 0.13
JBJS	5.9 ± 10.1	3.2 ± 5.6	3.8 ± 11.4	1.1 ± 1.5	2.7 ± 10.7	0.2 ± 0.4	0.03 ± 0.20
Total	4.6 ± 8.0	2.6 ± 4.5	3.6 ± 10.0	1.1 ± 1.3	2.5 ± 9.4	0.3 ± 0.6	0.03 ± 0.17

CORR = *Clinical Orthopaedics and Related Research*, JAAOS = *Journal of the American Academy of Orthopaedic Surgeons*, JBJS = *Journal of Bone and Joint Surgery*

**Figure 1:**
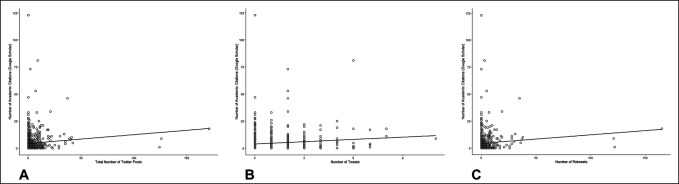
Graph showing scatter plots with regression lines showing the number of Google Scholar academic citation versus (**A**) total number of social media posts, (**B**) number of tweets, and (**C**) number of retweets.

**Figure 2:**
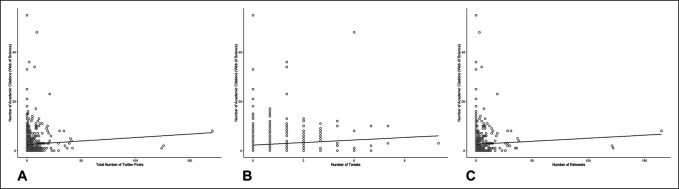
Graph showing scatter plots with regression lines showing number of Web of Science academic citation versus (**A**) total number of social media posts, (**B**) number of tweets, and (**C**) number of retweets.

The number of academic citations per article on Google Scholar was weakly correlated with the number of social medial postings (ρ = 0.10, *P* = 0.005, Figure 1, A), the number of tweets (ρ = 0.11, *P* = 0.002, Figure 1, B), and the number of retweets (ρ = 0.08, *P* = 0.03, Figure 1, C). Similarly, the number of academic citations per article on Web of Science was weakly correlated with the number of social medial postings (ρ = 0.09, *P* = 0.009, Figure 2, A), the number of tweets (ρ = 0.09, *P* = 0.007, Figure 2, B), and the number of retweets (ρ = 0.07, *P* = 0.04, Figure 2, C).

The number of official tweets had no significant correlation with academic citation on Google scholar (*P* = 0.4) or Web of Science (*P* = 0.2). Similarly, the number of author tweets had no significant correlation with academic citation on Google scholar (*P* = 0.2) or Web of Science (*P* = 0.5). *Post hoc* power analysis showed that a sample size of 835 had greater than 80% power in detecting a significant correlation coefficient of 0.1.

No significant difference existed in social medial postings (*P* = 0.09), the number of retweets (*P* = 0.1), or the number of author tweets (*P* = 0.7) among the three journals. However, the number of tweets (*P* = 0.04) and the number of official tweets (*P* < 0.0001) significantly differed among the three journals, with CORR demonstrating the highest social media utilization, followed by JBJS and JAAOS.

Twenty-eight citation discordant publications were identified. Eighteen publications had at least 10 times the academic citations than social media posts (five arthroplasty, three general interest, three oncology, two spine, two foot and ankle, two shoulder, and 1 pediatric). Nine publications had at least 10 times the social media posts than academic citations (three general interest, two arthroplasty, two trauma, 1 foot and ankle, and 1 shoulder). Comparison of citation discordant publications showed no difference in publishing journal (*P* = 0.9), but the publications with a discordantly high number of social media posts were published later (*P* < 0.0001).

## Discussion

The role of social media platforms in orthopaedic research has expanded in recent years. Both patients and surgeons increasingly look to social media platforms such as Twitter to communicate, educate, and disseminate information,^1,2,3,4,5,6^ and many academic journals have adopted Twitter profiles in response.^7,8^ In this study, through a systematic analysis of recent research publications in three high-impact orthopaedic surgery journals, we have shown that social media posting is weakly, but markedly, correlated with academic citation. Although each of the three journals demonstrated a presence on social media, the notable differences in social media utilization between the journals may represent an opportunity to more effectively share published research by leveraging online platforms.

Conventional academic citation of research publications is a long-standing and reliable metric of research impact and productivity and is clearly correlated with academic rank^11-13^ and research funding.^14^ However, there are disadvantages to conventional academic citations. The impact of primary research publications may be underrepresented if subsequent publications cite secondary sources or review articles.^9^ Moreover, academic citations often require many years to accrue, whereas social media mentions are often a more immediate measure of scholarly impact.^9,15^ Finally, conventional academic citations fail to capture the impact of research on readers who do not publish or cite themselves and therefore may not be the most complete metric of the societal impact of scholarly works. For this reason, some authors argue that alternative metrics, such as social media mentions, should be considered when measuring scientific impact.^15,16^

Our findings are in agreement with the previous literature. Evaniew et al^9^ performed a systematic review of randomized controlled trials of musculoskeletal conditions published between 2011 and 2014 and found a median of two online postings per research publication and a weak significant correlation with academic citation. Similarly, Kunze et al^17^ studied conventional academic citations and alternative metrics of original research in five high-impact orthopaedic surgery journals in 2016 and found a weak association between the Altmetric Attention Score and citation rate. Our results support a positive relationship between social media posts and academic citations of orthopaedic research; however, although adoption of social media platforms continues to increase among researchers and journals, the strength of correlation between social media posts and academic citations has not increased. Moreover, social media utilization differs markedly across journals, and official tweets are a notable driver of this difference. As expected, we have shown that longer time since publication is correlated with a greater number of academic citations. However, this temporal relationship was not demonstrated with social media posts, which supports the theory that social media dissemination of research occurs more rapidly after publication and is not subject to the same time delay as conventional citations.

There are limitations to this study. First, the study focused on research publications from 2018 to 2019. Given the rapidly evolving adoption of social media, we confined our study to these recent years to best capture current trends; however, the number of conventional academic citations will likely continue to grow in the years to come. Second, only three orthopaedic surgery journals were studied. Although these three journals are certainly not exhaustive of the orthopaedic literature, high-impact journals were chosen, and more than 800 publications were included, which we believe is an accurate representation of the current orthopaedic landscape. Third, there are questions pertaining to the social media posts of orthopaedic surgery research publications that are outside of the scope of this study. We are unable to comment on the content, quality, and accuracy of social media posts because these clearly do not undergo the peer-review process inherent to academic citations. Although we have found that more recent publications are more likely to accrue a discordant number of social media posts, our study was not designed to identify factors that make a publication “go viral” onametrics of online research dissemination, such as the number of news mentions, downloads, or shares, are difficult or impossible to quantify.

Social media platforms are increasingly used and effective means of sharing original research and have been largely adopted in orthopaedic surgery. Social media mentions represent a more rapid and, in some ways, a more equitable assessment of the societal impact of research than conventional academic citations. We have demonstrated a weak but significant correlation between social media posts of recent orthopaedic surgery research publications and conventional academic citations. Utilization of social media varies between journals and between authors, which may represent opportunities to leverage online platforms to more effectively share novel research findings.

## References

[1] Haeberle HS Egger AC Navarro SM: Social media and pediatric scoliosis: An analysis of patient and surgeon use. Surg Technol Int 2017;31:189-196. 29020706

[2] Klietz ML Kaiser HW Machens HG Aitzetmüller MM: Social media marketing: What do prospective patients want to see? Aesthet Surg J 2020;40:577-583. 31361806 10.1093/asj/sjz204

[3] Lander ST Sanders JO Cook PC O'Malley NT: Social media in pediatric orthopaedics. J Pediatr Orthop 2017;37:e436-e439. 28719545 10.1097/BPO.0000000000001032 PMC5642967

[4] Ramkumar PN Navarro SM Cornaghie MM: Social media in shoulder & elbow surgery: An analysis of twitter and instagram. Int J Sports Med 2018;39:564-570. 29758568 10.1055/s-0043-124369

[5] Rozental TD George TM Chacko AT: Social networking among upper extremity patients. J Hand Surg Am 2010;35:819-823.e1. 20227837 10.1016/j.jhsa.2009.12.043

[6] Varady NH Chandawarkar AA Kernkamp WA Gans I: Who should you be following? The top 100 social media influencers in orthopaedic surgery. World J Orthop 2019;10:327-338. 31572669 10.5312/wjo.v10.i9.327 PMC6766466

[7] Asyyed Z McGuire C Samargandi O Al-Youha S Williams JG: The use of twitter by plastic surgery journals. Plast Reconstr Surg 2019;143:1092e-1098e. 10.1097/PRS.0000000000005535 31033839

[8] Hughes H Hughes A Murphy C: The use of twitter by the trauma and orthopaedic surgery journals: Twitter activity, impact factor, and alternative metrics. Cureus 2017;9:e1931. 29464138 10.7759/cureus.1931 PMC5807027

[9] Evaniew N Adili AF Ghert M: The scholarly influence of orthopaedic research according to conventional and alternative metrics: A systematic review. JBJS Rev 2017;5:e5. 10.2106/JBJS.RVW.16.00059 28557819

[10] Twitter Announces First Quarter 2019 Results. Twitter, Inc, 2019 https://s22.q4cdn.com/826641620/files/doc_financials/2019/q1/Q1-2019-Earnings-Release.pdf. Accessed March 29, 2020.

[11] Bastian S Ippolito JA Lopez SA Eloy JA Beebe KS: The use of the h-index in academic orthopaedic surgery. J Bone Joint Surg Am 2017;99:e14. 28196042 10.2106/JBJS.15.01354

[12] Ence AK Cope SR Holliday EB Somerson JS: Publication productivity and experience: Factors associated with academic rank among orthopaedic surgery faculty in the United States. J Bone Joint Surg Am 2016;98:e41. 27194503 10.2106/JBJS.15.00757

[13] Lopez J Susarla SM Swanson EW Calotta N Lifchez SD: The association of the H-index and academic rank among full-time academic hand surgeons affiliated with fellowship programs. J Hand Surg Am 2015;40:1434-1441. 26026351 10.1016/j.jhsa.2015.03.026

[14] Stavrakis AI Patel AD Burke ZD: The role of chairman and research director in influencing scholarly productivity and research funding in academic orthopaedic surgery. J Orthop Res 2015;33:1407-1411. 25940983 10.1002/jor.22919

[15] Thelwall M Haustein S Larivière V Sugimoto CR: Do altmetrics work? Twitter and ten other social web services. PLoS One 2013;8:e64841. 23724101 10.1371/journal.pone.0064841 PMC3665624

[16] Pulido CM Redondo-Sama G Sordé-Martí T Flecha R: Social impact in social media: A new method to evaluate the social impact of research. PLoS One 2018;13:e0203117. 30157262 10.1371/journal.pone.0203117 PMC6114920

[17] Kunze KN Polce EM Vadhera A: What is the predictive ability and academic impact of the altmetrics Score and social media attention? Am J Sports Med 2020;48:1056-1062. 32109148 10.1177/0363546520903703

